# Functionalization of Textile Materials for Advanced Engineering Applications

**DOI:** 10.3390/ijms27062708

**Published:** 2026-03-16

**Authors:** Andrey A. Vodyashkin, Mstislav O. Makeev, Dmitriy S. Ryzhenko, Anastasia M. Stoynova

**Affiliations:** 1 Bauman Moscow State Technical University, 2-я Baumanskaya St., 5, Moscow 105005, Russia; 2 Institute of Pharmacy and Biotechnology, RUDN University, Miklukho-Maklaya Str. 6, Moscow 117198, Russia; stoynova-am@rudn.ru

**Keywords:** textiles, fabric, surface modification, fabric functionalization, conductive textiles, bioactive textiles, stimulus-sensitive materials

## Abstract

Textile materials represent a versatile class of engineering substrates widely used in apparel, domestic products, and medical protective systems. Despite their extensive application, large-scale textile production has seen limited integration of fundamentally new functionalization strategies. In recent years, however, advances in materials science have enabled the development of textiles with tailored electrical, adaptive, and biological functionalities. This review summarizes recent progress in the functionalization of textile materials with a focus on approaches relevant to engineering and industrial implementation. Particular attention is given to conductive textiles designed for operation under extreme environmental conditions, including low-temperature climates. Methods for integrating electrically conductive elements into fibrous structures are discussed, highlighting their potential for sensing, thermal regulation, and energy-related applications such as powering portable electronic devices. Inkjet printing is presented as a scalable technique for high-resolution deposition of conductive patterns while preserving the mechanical integrity and aesthetic properties of textile substrates. In addition, adaptive and stimuli-responsive textile systems are reviewed, including materials capable of responding to thermal, optical, or chemical stimuli, with applications in camouflage, wearable systems, and multifunctional surfaces. The review further addresses the development of bioactive textiles, emphasizing antibacterial functionalization using organic and inorganic agents to mitigate the spread of pathogenic microorganisms. The relevance of such materials has been underscored by recent global viral outbreaks. Overall, this work aims to provide a materials science perspective on emerging textile functionalization strategies and to facilitate the transition of these technologies from laboratory-scale research to practical engineering applications.

## 1. Introduction

Textile materials play a pivotal role in both everyday life and industry [1,2]. They can be used to create garments [3], footwear [4], furniture components [5], personal protective equipment [6] sporting goods [7], etc. The transition towards functional and versatile textiles has the potential to markedly enhance the quality of life for individuals [8,9]. This phenomenon may be attributable to the enhanced comfort afforded by the garment, as well as its potential to provide greater autonomy by reducing dependence on external power sources [10,11], as well as enhancing safety in the event of natural phenomena and natural disasters [12,13]. The contemporary imperative for textiles to exhibit functionality, environmental sustainability and safety has given rise to the development of novel methodologies for their modification and functionalisation [14,15]. Notwithstanding the considerable progress that has been made in this field, challenges persist with regard to preserving the fundamental properties of fabrics while incorporating new functions and ensuring that manufacturing processes are both cost-effective and environmentally sustainable [16,17].

The creation of functionalised textile materials can take place during the production of fibre (yarn) or post-production methods that treat fabrics that have already been finished. Modification in fibre production [18] enables the creation of flame retardant, hydrophobic and other specialised fibres and yarns [19,20,21]. However, it should be noted that these materials may be subjected to a range of treatments, including thermal, mechanical, chemical and imaging processes, during the manufacturing of the final products. These treatments have the potential to reduce the effect of the original treatment [22].

In this regard, it is imperative to modify finished fabrics that are to be further subjected to minimal impact, thereby optimising the functionality of the final product [23].

A plethora of strategies for the functionalisation of textile materials have been posited, encompassing the following:

- Improved colorimetric performance of the final product [24];
- Creation of light-absorbing and reflective textile materials [25];
- Improved strength and wear resistance [26];
- Development of hydrophobic textile materials [27,28];
- Increased air and moisture permeability [29,30];
- Improvement of fire resistance [31,32];
- Development of adaptive textile materials [33];
- Development of multifunctional composite textile materials [34];
- Development of bioactive textile materials [35,36];
- Development of personal protective equipment for extreme conditions [37];
- Development of electrically conductive textile materials [38,39].

It is important to note that not all directions have real prospects of implementation in industry and mass production. This is due to high cost, sharp change in fabric properties and complexity of modification conditions [40,41].

One of the most common methods of textile functionalisation is the surface modification of fabrics [42,43].

This process is typically carried out either by applying a solution to the fabric’s surface or by fully immersing the fabric in the solution [44,45]. During this process, covalent bonds form between the fabric’s functional groups and the modifiers, accompanied by intermolecular interactions, leading to a marked change in the material’s properties.

A variety of substances, encompassing both small molecules [46] and polymers [47] as well as nanomaterials [48], are employed as modifying agents. Combinations of these agents can also be used to improve the performance, as demonstrated in the following studies: [48,49,50]. Considering the large volumes of textile production, potential modifiers should have low cost, high biosafety and environmental friendliness. The absence of the need for complex equipment and time-consuming, high-energy processes (e.g., annealing) will significantly increase the chances of successful integration of the method into the light industry [51,52].

The utilisation of textile materials with augmented functional properties has the potential to serve as the foundation for the development of smart materials in the future. These materials are poised to enhance the quality of life for individuals by facilitating ease, comfort, and safety [53,54]. As demonstrated in the following sources, which can be accessed via the provided links, conductive elements with sensors can be used to protect against natural phenomena and natural or man-made disasters [55,56]. In addition, they can be used to increase comfort and supplement human heating or as a source for charging mobile devices [57]. Furthermore, conductive elements with sensors can present unique systems for monitoring human physiological parameters [58,59,60].

The creation of bioactive textiles has the potential to contribute to the reduction in the spread of diseases and the maintenance of human health [61,62,63].

## 2. Basic Research Strategy and Methodology

In this review, the most promising areas of textile materials modification were identified. The presentation encompassed contemporary methodologies employed in the fabrication of functional materials, accompanied by a discourse on their respective merits and constraints pertinent to their industrial implementation. Universal characterisation methods applicable for material quality assessment and colorimetric characteristics of images applied to textiles are also reviewed. The primary industry outlook anticipated in the forthcoming years is hereby presented. The creation of this review involved the utilisation of a systematic literature review, which was employed to examine research articles, patents and books that would describe the main strategies and approaches to modify textile materials. The search was conducted using the well-known databases Google Scholar, Dimensions, ScienceDirect, and PubMed (for the section on bioactive materials).

**Figure 1:**
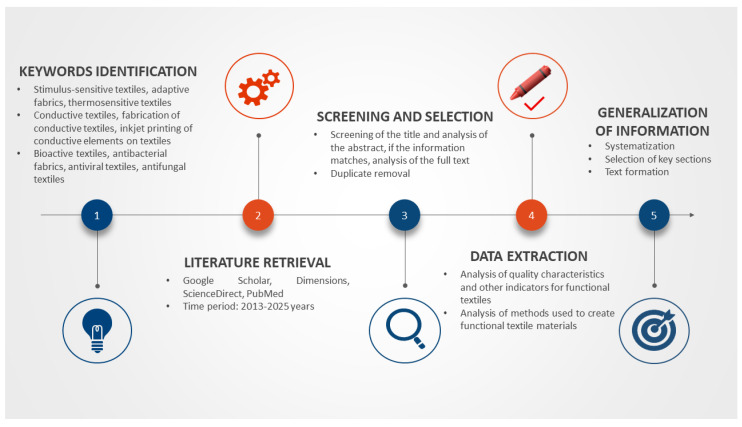
Mechanism for selecting and screening information used for review.

The selection of targeted and relevant information was based on a literature search using words found in article titles, abstracts and keyword lists. The analysis was conducted exclusively on English-language articles that had been published in peer-reviewed academic journals. To provide a comprehensive overview of the most promising and innovative developments in textiles, a comprehensive review of publications from 2013 to 2025 was conducted. This will enable the reader to familiarise themselves with the main trends and breakthrough methods of modifying textile materials. However, in order to elucidate certain fundamental aspects, such as fibre and fabric manufacturing methods or the classification of textile materials, earlier publications have been consulted to provide the original, more comprehensive information. Figure 1 illustrates the comprehensive process of literature selection and analysis undertaken for the present article.

## 3. Textile Fibres, Methods of Modification. General Stages of Production of Textile Items

### 3.1. Research Trends in the Textile Industry

**Figure 2:**
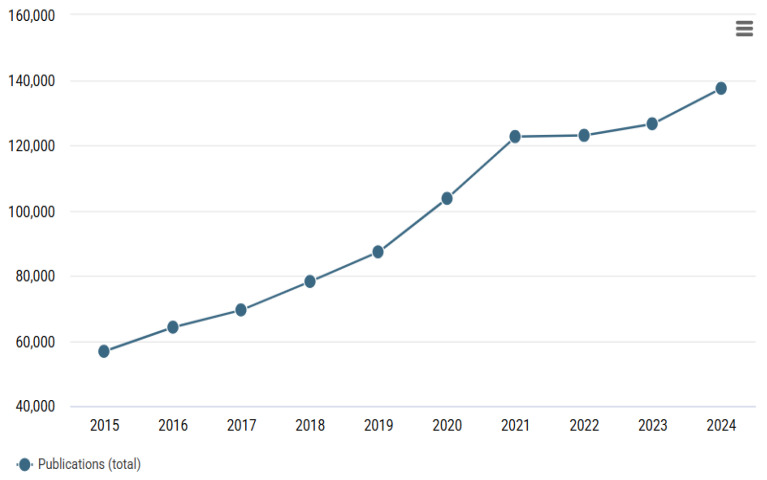
Number of publications by year for the query ‘textile’ (Dimensions data).

**Figure 3:**
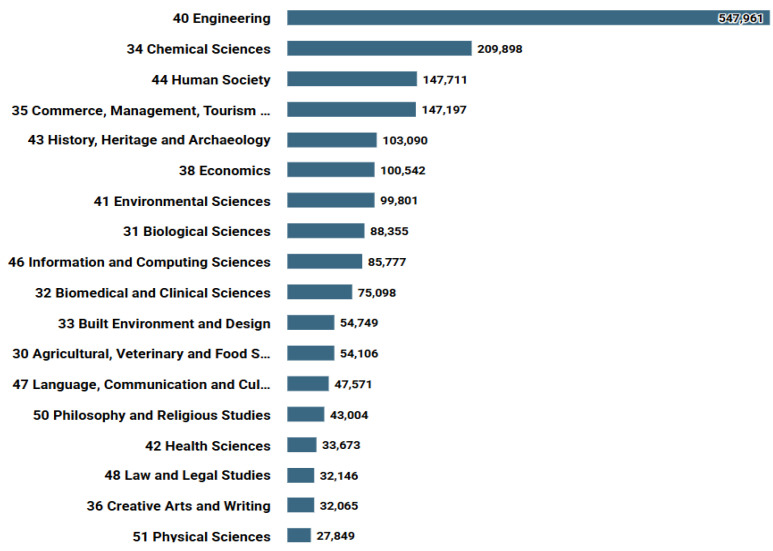
Main categories of research on the query ‘textile’ (Dimensions data).

Textiles have long been recognised as playing a pivotal role across a wide range of human activities and endeavours, thus garnering the sustained interest of scholars across various disciplines [64]. This has resulted in an annual increase in research activity in this field, as evidenced by the data presented in Figure 2. The development and research of these areas has the potential to exert a significant impact on various sectors of industry [65]. The integration of functional materials has the potential to streamline various industries, enhancing their ergonomics and safety [66]. As illustrated in Figure 3, the primary categories of research encompassing textile materials are evident. The categories reflect the main trends and promising directions in the field of textile materials. As is evident, the initial two categories pertain to engineering and chemical sciences. Consequently, the development of novel modification methods and the creation of new textile materials is a highly promising avenue for research [67,68]. A further pivotal category is that of management and economics. The cost of textiles is a determining factor in their production, given that they are large-tonnage products [69,70]. It is vital to acknowledge that a synthesis of quality (durability), functionality and cost is pivotal in order to ensure that the product is rendered most attractive to the consumer [71,72]. The recent prevalence of biological and medical textile materials is indicative of prevailing trends in the field [73,74]. These materials have two distinct applications: firstly, they can be used as personal protective equipment; secondly, and more pertinently, they can be incorporated into a person’s everyday attire [75].

In recent years, there has been an increasing emphasis on materials that have the capacity to protect health, or to maintain an environment that is conducive to health [76,77]. It is an irrefutable fact that the biomedical sector has seen a marked increase in activity since 2020, coinciding as this does with the advent of the pandemic caused by the SARS-CoV-2 virus (COVID-19) [78,79]. A significant corpus of literature has been published in the ecological direction. It is pertinent to observe that this pertains to the promising direction of recycling and reuse of materials used for textile products [80,81,82]. The potential for this kind of work is twofold: on the one hand, it can contribute to the establishment of closed-loop industries, and on the other, it can play a role in reducing the environmental impact on our planet.

### 3.2. Classification of Fibres Used in the Textile Production

Textiles are defined as inhomogeneous anisotropic materials whose properties are contingent on the fibres from which they are composed, the manner in which they are modified, the spinning process and the method of manufacturing a particular textile product [83]. The fibres constitute the initial component and can significantly influence the eventual properties of the final material [84,85]. The fibres employed within the textile industry can be categorised into the following groups:

(1) Naturally occurring
  (a) Materials of plant origin include cotton (one of the most common textile materials), linen, bamboo, hemp and jute, amongst others. Furthermore, the fibres utilised in the fabrication of textile materials can also be derived from chemicals extracted from the same materials.
    In addition to the utilisation of vegetable raw materials in their purest form, the extraction of chemicals from these materials can be employed for the manufacture of textile materials. The majority of these substances are polymers, including cellulose (and its derivatives), alginates, and certain types of proteins.
  (b) The material under discussion is of animal origin, and examples of such materials include silk and wool. A range of animal sources, including but not limited to sheep, camels, horses and hares, have been identified as potential sources of hair and wool.
(2) Synthetic. The range of artificial materials encompasses polyester, aramid, acrylic polymers, polyamide, polypropylene, polyethylene, and numerous others
(3) Mineral substances include asbestos, glass fibre and carbon fibre. Their utilisation is limited to special protective clothing. Mineral materials are not used as a basis for textiles; however, they can be used as covering or doping elements, and can also be used as protective fragments. These fragments can increase mechanical strength or resistance to high-temperature effects.

### 3.3. Production and Modification of Fibres

Fibres are produced through two fundamental processes: isolation and synthesis. Isolation entails the separation of fibres from plant or animal raw materials. Synthetic fibres are produced through a different process of synthesis [86,87]. In general practice, special treatments may be applied to extract fibres from plant raw materials, and these may involve physical or mechanical treatments [88] as well as chemical [89,90]. Moreover, there exist methodologies which involve the utilisation of biological entities, such as enzymes [27,91]. It is acknowledged that the application of additional treatments to fibres can yield a number of benefits, including enhanced properties and characteristics [92]. Additional processing can improve mechanical, thermal properties, functionalise the fibre with specific groups, and customise the surface properties [93,94,95]. It is generally accepted practice to apply the treatment to natural fibres. This is due to the fact that the parameters of synthetic fibres are more amenable to control and are not contingent on the raw material, but rather on the manufacturing and moulding conditions. The principal agents employed for the modification of textile fibres are enumerated in Table 1.

Physical methods, including ozonation, plasma, and temperature treatments, can be employed to enhance structural and morphological properties. Targeted grafting of specific groups can also be conducted [112,113]. It has been established that fluorocarbons, isocyanates, and enzymatic preparations can be employed for the treatment of certain fibre types, with natural fibres being a notable example [114,115].

It is important to note that any modification to the fibres will have a significant impact on the cost of producing the final product. However, further processing and manufacturing of the final product may serve to mitigate the effect of this process.

### 3.4. The Main Stages of Manufacturing Textile Materials

The selection of a specific type of raw material (fibres) is a highly individualised process for each manufacturer, contingent on social, economic, aesthetic and resource factors, as well as the intended application of the finished product [116,117,118]. In recent years, there has been an increased focus on enhancing the mechanical and aesthetic properties of fabrics. This has led to the emergence of mixed fabrics, which are composed of vegetable raw materials, such as cotton, and contain small quantities of artificial fibres, such as polyester [119,120,121,122].

The manufacturing process of fabrics from synthetic and natural materials comprises standardised procedures that have been utilised in the textile and light industry for a considerable duration [123]. Industrialisation, digitalisation of industrial processes and, in recent years, the integration of artificial intelligence [124] help to optimise, speed up procedures, and replace manual labour with machine labour, but key steps remain unchanged [125,126]. The production of textile fabrics is comprised of several key stages, namely: fibre production and modification, fibre yarn manufacturing, fabric/non-woven fabric manufacturing, and fabric finishing and dyeing where necessary.

The separation and modification of the fibres is followed by the yarn production step. This process is accompanied by the production of yarns from one or different fibre types on special machines [127]. It is evident that spinning can be categorised into two distinct types: namely, staple spinning and continuous spinning [128]. The selection of optimal yarn manufacturing procedures and types is contingent upon the type of raw materials and production resources employed. The yarn’s cost constitutes a substantial proportion of the overall expense associated with the final product [129]. As demonstrated in the relevant literature, a thorough investigation has been undertaken of the spinning procedure, along with its immediate prospects [130,131,132,133].

The next stage directly is the manufacture of fabrics, nonwovens, webs, felts, etc [134]. The process of making and moulding fabrics from yarn is a multi-step procedure, the essence of which is the interweaving of two systems of threads (yarns) perpendicular to each other [135]. The selection of a scheme (pattern) of yarn weave is contingent on the composition of the yarn, the purpose of the end yarns, the strength of the twist and other factors [136]. The main methods of making are weaving, knitting or plaiting [137].

The final stage in the manufacture of textile materials is the finishing of the fabric [138,139]. The desired outcome can be achieved through a variety of methods, including but not limited to bleaching, printing, dyeing, and functionalising textiles [140,141,142].

The application of dyes to textiles composed of both artificial and natural fibres can occur during various stages of the textile manufacturing process. However, following the completion of the manufacturing process, the material will exhibit a diminished degree of susceptibility to colour change and will retain its colour characteristics [143,144]. The study examined the relationship between colorimetric characteristics and colour intensity on the aesthetic perception of finished products and found them to be key factors in this regard [145]. The utilisation of dyes for the dyeing of fibres is contingent upon the fibre’s composition, wherein different dyes, encompassing both organic and pigmented varieties, may be employed [146,147]. It is imperative that the textile material exhibits a high degree of adhesion to the dye, whilst also preserving its functional and mechanical properties following the dyeing process [148,149].

In addition to the application of dyes, patterns and images can be incorporated into textiles, thereby conferring a distinctive characteristic upon this class of garment [150,151,152].

The functionalisation of textile materials has been shown to offer protection against a variety of potential hazards, including fire, water, and biological objects. Furthermore, this process can lead to an increase in the specific surface area of the material, resulting in the creation of a porous or sorbent substance. The subsequent sections will provide a more detailed exposition on the promising areas of functionalisation, along with the fabrication and control methods.

## 4. Conductive Textile Materials

The utilisation of textile materials incorporating conductive components has the potential to transform the future of clothing, enabling the integration of functional elements such as charging capabilities for electronic devices and the incorporation of heating mechanisms within textiles. This advancement not only enhances the versatility of contemporary textiles but also paves the way for the development of advanced “smart” textile materials.

The employment of flexible guiding patterns on textiles is imperative for the creation of sophisticated devices on clothing and for the integration of smart textile materials into everyday life. These elements can be incorporated into a textile material or applied to its surface in a comprehensive manner. Numerous technologies are currently employed for the application of conductive coatings, thereby enabling the attainment of continuous conductive coatings through the utilisation of magnetron sputtering (reactive magnetron method) [153,154], spraying at ambient temperature [155], high temperature thermal deposition [156], multilayer deposited passivation [157], amongst others. The creation of transparent conductive mesh structures is also possible through photolithography methods [158], self-organisation [159,160], and other methods. In the majority of cases, these coatings exhibit superior wear resistance to bending, with comparable or even superior characteristics in terms of transparency and surface resistance [161]. The integration of conductive elements within textile materials has emerged as a pivotal area of research, with significant potential for the development of novel sensors and smart clothing components. This integration facilitates the enhancement and creation of these components, as evidenced by the research published in [162].

### 4.1. Inkjet Printing for the Creation of Conductive Textile Materials

Conductive layers can be applied to fabrics by means of inkjet printing. It is evident that conventional ink formulations employed in the production of conductive inks frequently entail the utilisation of toxic solvents and additives. These substances introduce complexity to the manufacturing process, impede compatibility with printer hardware, and diminish device performance. It is important to note that even water-based inks containing conductive polymers, carbon nanotubes, graphene or metallic nanoparticles require additional dispersants, co-solvents and difficult conditions to obtain sprayable colloidal suspensions that can be successfully applied to the surface of materials. It is important to acknowledge the current limitations of inkjet printing methods, which are restricted to the application of conductive components to textiles. Concurrently, they demonstrate considerable promise in terms of their ability to streamline conventional methodologies and enhance the environmental sustainability of the application of conductive materials to textile surfaces. Furthermore, advancements in inkjet printing technology have enabled the enhancement of print resolution, thereby facilitating the more precise reproduction of fine details.

Felix Hermerschmidt and others the feasibility of utilising inkjet printing as a technique for fabricating conductive electrodes has been demonstrated with notable success [163].

Silver nanoparticles have become a prevalent material in the creation of conductive layers on textiles [164,165,166]. The simplicity of the methods of obtaining and high colloidal stability [167] of these nanoparticles has led to their widespread use [168]. Furthermore, the methods used to obtain these nanoparticles are also highly versatile due to their ease of use. The breadth of their potential applications is evident in the extensive list of sources which can be found in the following [169].

A. Boumegnane et al. proposed a method for synthesising starch-stabilised silver nanoparticles, which were then added to ink for use in inkjet printing. The nanoparticles were successfully printed on textile materials. The authors demonstrated the effect of the fixing temperature on the final resistance of the patterns [170]. Wei Li et al. developed inks based on silver nanoparticles and a mixture of glycerine, ethanol and ethylene glycol, which were used to regulate viscosity and surface tension. The authors demonstrated the possibility of creating conductive patterns on a wide range of surfaces, from PDMS to photographic paper. Furthermore, Wei Li et al. corroborated the feasibility of utilising these inks in conventional office printers, thereby markedly enhancing the printing velocity of conductive patterns and potentially streamlining the incorporation of methodologies within industrial contexts [171].

The utilisation of inkjet printing on textiles with the incorporation of silver nanoparticles has been previously explored in a number of studies. Please refer to the following documentation for further details: [172,173,174].

At present, there are a number of issues that act as obstacles to the large-scale manufacture of textile materials with conductive patterns. Direct printing on fabrics is not possible due to the complex composition of the ink (necessary to enable inkjet printing). This composition prevents the creation of a continuous conductive path without the printing of multiple layers [175]. Moreover, the implementation of inkjet printing on textile materials gives rise to several technical challenges. Primarily, the process of fabricating continuous high-conductivity tracks on coarse fabric with a thin layer is arduous. Additionally, the majority of fabrics demonstrate an inability to withstand prolonged curing, and their resistance to stretching and bending is limited [176]. A number of key features and specifics of ink for conductive patterns are presented in the works. The following doi references can be used to access the relevant literature: [177,178,179].

**Figure 4:**
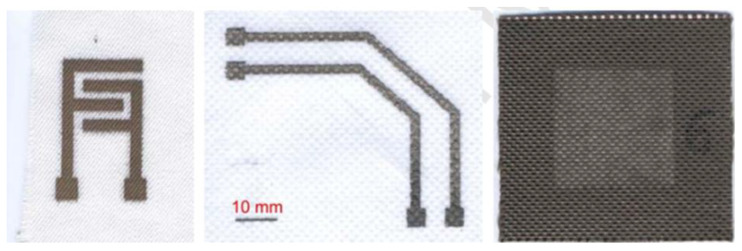
Conductive images printed using silver nanoparticles on various materials [180].

In the work of Z. Stempien et al., silver nanoparticles filtered with a size of less than 200 nm were used as a component for conductive ink. The ReaJet SK 1/080 printer (Mühltal, Germany) was utilised for the printing process, boasting a bitmap resolution of 100 dots per inch. Prior to the printing process, a pre-coating consisting of an acrylate resin was applied with the objective of mitigating surface roughness. Consequently, a series of images were produced (Figure 4) that exhibited conductivity. The fractions of silver nanoparticles were confirmed as successful by electron microscopy and EDX analysis, which demonstrated the pure silver phase. The authors conducted a study of the surface resistance, which varies with increasing number of silver layers for all textile substrates. Following the application of two layers of silver to the PP nonwoven fabric, a distinct conductivity was observed. The nature of this conductivity was found to be dependent upon the type of textile material used. It is noteworthy that all the samples obtained were flexible and exhibited high resistance to both washing and dry cleaning [180].

**Figure 5:**
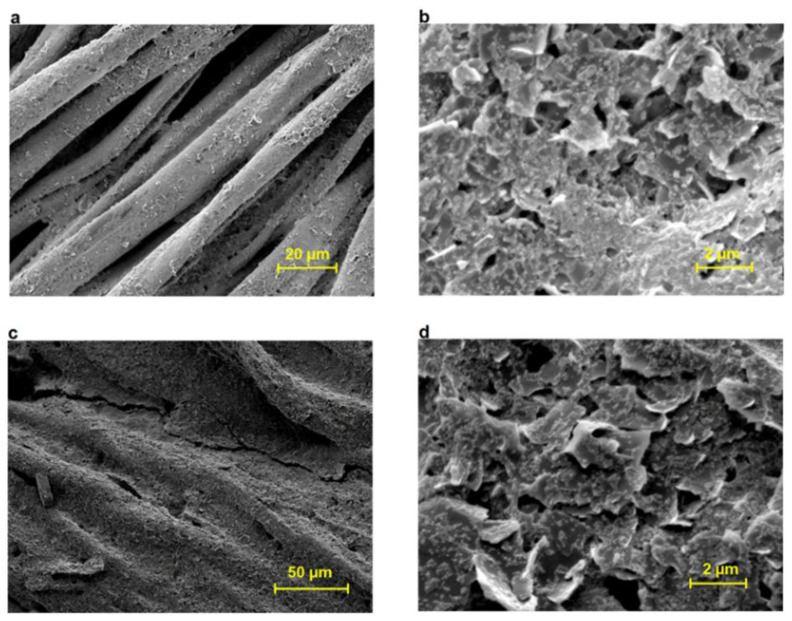
Micrographs of cotton fabric for (**a**,**b**) untreated, (**c**,**d**) processed samples [182].

Carbon micro- and nanomaterials have emerged as a particularly promising solution for the integration of conductive components into clothing. The utilisation of metal and graphene nanoparticles in conjunction has been demonstrated to yield a synergistic effect, thereby enhancing the properties of the conductive layer. In a recent study, Nazmul Karim and his colleagues demonstrated a significant enhancement in the conductivity of paper and cotton materials through the incorporation of silver nanoparticles. This enhancement was observed when the amount of silver nanoparticles added to the inks ranged from 1 to 3% by weight. In their work, the authors optimised the ink fixing temperature, which exerts a pivotal effect on the conductivity of the layer of the final product. In addition to the workplace context [180], the authors draw attention to a substantial decline in resistance concomitant with an augmentation in the number of layers. The effectiveness of preliminary surface modification using styrene/divinylbenzene nanoparticles is demonstrated, with the particles being used to increase surface free energy and reduce roughness [181]. It has been demonstrated [182] that the conductive layer can exhibit a high degree of resistance to bending and folding while maintaining constant resistance, demonstrating significant potential for the creation of hybrid materials that can be effectively used in the textile industry over a long period of time (Figure 5). Such materials can maintain functionality throughout the entire service life of the material.

Dual metal/carbon nanoparticle systems have been actively used as inks to create conductive patterns on textiles [183,184,185,186,187].

**Figure 6:**
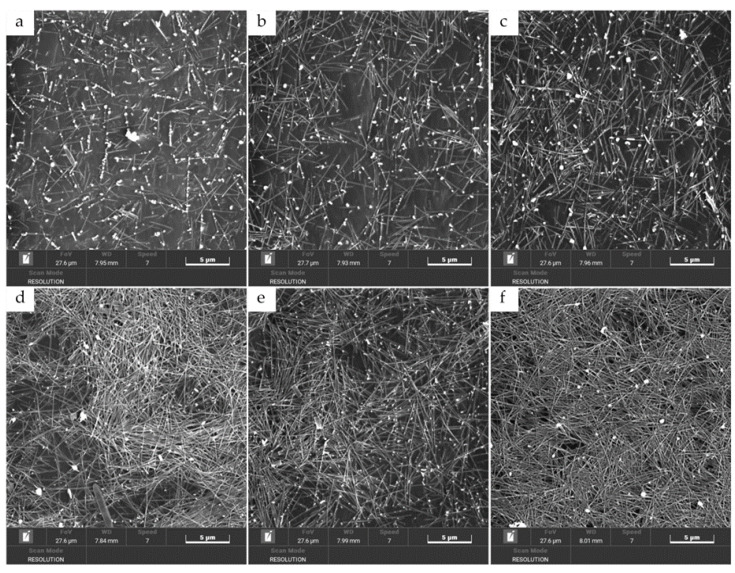
The effect of the number of silver nanowire layers on the morphology of the film surface/ SEM images of AgNWs films with the printing layers of 3 (**a**), 6 (**b**), 9 (**c**), 12 (**d**), 15 (**e**), and 18 layers (**f**), respectively [188].

In their paper, Yuehui Wang and colleagues propose a method for creating conductive ink based on silver nanowires (Figure 6) [188]. In the course of their work, the authors optimise the printing conditions (number of layers, surface temperature, silver nanowire parameters) to create films with an average surface resistance of 13 Ω/square −1 and a high degree of transparency.

The articles presented herein demonstrate the potential use of inkjet printing for textile modification, which can be used to present strategies for creating smart materials, antennas, sensors, and so forth. A substantial body of research has been conducted on the possibility of creating conductive patterns on the surface of a material by adding metal or carbon nanoparticles to the ink. The application of inkjet printing technology facilitates the transfer of patterns onto textiles with a high degree of resolution, thereby ensuring the preservation of intricate details. This process results in the creation of unique conductive images. In certain instances, pretreatment of the material and printing of multiple conductive layers may be necessary to achieve the desired outcome and mitigate resistance. The majority of the technologies utilised in this context are those employed in laboratory settings. However, when transitioning to industrial scales, the proposed methodologies may encounter a range of technological scaling challenges. This suggests a significant requirement for the development of versatile and economical formulations that can enhance the process, accelerating it and reducing costs.

### 4.2. Alternative Methods for the Creation of Conductive Textile Materials

Inkjet printing has emerged as a novel and promising method for the fabrication of conductive patterns. However, its translation into commercial applications and large-scale production remains challenging, thus hindering its integration into existing technological frameworks [189].

A method for creating conductive textile polyacrylonitrile webs has been proposed by Md Abdullah Al Faruque and others. A conductive agent composed of 1% graphene oxide was applied using the brush rubbing and drying method. Furthermore, GO-coated tissues were treated with hydrazine vapours to restore the material. The utilisation of electron microscopy has substantiated the uniform distribution of GO (graphene oxide) sheets, a finding of paramount importance for augmenting conductivity on the textile surface. The colour intensity of the materials, both with and without the restoration procedure, was increased, while the colour of the material changed to a darker one, which is due to the inclusion of GO in the fabric matrix. The recovered tissues demonstrated a surface resistance of approximately 285 ± 1.72 ohms, and an electrical conductivity of approximately 0.35 ± 0.78 S/cm. Concurrently, the incorporation of 25% wool into the polyacrylonitrile matrix has been demonstrated to enhance electrical conductivity to approximately 1.67 ± 0.83 S/cm. This phenomenon may be attributed to an augmented interaction between the graphene oxide sheets and the amino groups present in wool [190].

Potential agents include systems based on D-glucaric acid (GA)/chitosan and single-layer carbon nanotubes. In this case, the conductive composition can be applied to the surface by spraying or creating a pattern. Alternatively, a polymer can be included in the matrix during spinning to produce a solid, conductive textile [191].

Donghong Wang et al. presented a universal approach to creating conductive textile materials. Porous sponges, cellulose fibres and fabrics can all be used as a base. The method involves applying the sensitiser [SnCl_4_]^2−^ to the surface, followed by the catalyst [PdCl_4_]^2−^. The second stage involved metallising the surface using solutions containing metal ions. Nickel, cobalt, copper and silver were used to modify textiles. The electrophysical properties of the textiles depended on the application conditions and the type of metals deposited on the surface [192]. The paper also presents a similar strategy for modifying textiles with silver nanoparticles [193].

Conductive layers on textiles have the potential to function as flexible universal sensors. In a significant development, Hwajoong Kim and his team have successfully engineered a highly adaptable sensor, utilising silver nanoparticles. This breakthrough underscores the potential for the development of devices for the monitoring of diverse metabolic parameters, with the foundation being decorated fibres [194].

The paper proposed strategies for modifying cotton threads for the embroidery of conductive patterns on textiles. The pre-treated double-layer cotton thread was subjected to a process of cleansing, involving the removal of water-soluble and oily dressings. This was followed by a further bleaching procedure, which involved the use of a solution composed of sodium hypochlorite and sodium hydroxide. In order to modify the thread, it was treated with an aqueous solution of poly(3,4-ethylenedioxythiophene polystyrene sulfonate) (PEDOT:PSS), which additionally contained 3% ethylene glycol and 5% divinyl sulfone. The composition was applied using a developed stand-alone installation, which comprised four containers of 5 mL capacity. Each container contained a modifying compound, and a thread was stretched through them at a speed of 3.6 m/min. The modification of the composition was found to optimise the reduction in the necessity for embroidery on cotton, from 3.3 mOhm/cm to 40 mOhm/cm. This is associated with mechanical abrasion during the drawing process. The present paper proposes a methodology for the fabrication of conductive filaments that eschews the utilisation of metallisation and carbon materials. The technology has been demonstrated to be both cost-effective and straightforward to manufacture, thus facilitating its integration into extant technological frameworks [195].

Laser engraving has also been demonstrated as a successful method for the preparation of conductive layers and patterns on the surface of textiles [196]. The application of this technique involved the utilisation of a direct method of exposure to a Yb laser, with a duration of 255 femtoseconds and wavelengths of 1035.67 nm and 347.8 nm, which resulted in the generation of the third harmonic. It is important to note that the laser processing method was carried out in indoor conditions, which can facilitate the transfer of technology to enterprises. The resulting pattern exhibited substantial electrical conductivity, characterised by a layer resistance of 2.86 ohms/cm.

Amina L. Mohamed presented a strategy for the creation of conductive textiles, utilising titanium dioxide, polypyrrole (PPy), and organosilicon nanoparticles. The fabrication of TiO_2_/PPy nanoparticles has been utilised in the production of textiles. In order to achieve this objective, an aqueous solution of pyrrole was prepared, to which ammonium persulfate and titanium isopropoxide were added. The mixture was then left to stand for a period of 2 days. Subsequently, the particles were subjected to modification using 3-aminopropyltriethoxysilane in an aqueous medium at 80 °C, containing epichlorohydrin. In order to apply the composition to the fabric, bleached cotton fabric was immersed for 30 min in chloroform containing 2% nanoparticles. This was then kept for 48 h in a 5% aqueous NaOH solution for the hydrolysis of alkoxy silane groups. The utilisation of both TiO_2_/PPy nanoparticles and siliconized samples was employed in the experimental setup. The fabrics were subjected to a washing, drying and curing process at a temperature of 140 °C. Scanning electron microscopy was utilised to verify the uniform distribution of nanoparticles across the surface, while negligible aggregation of particles was observed in samples containing silicon. The modification resulted in a change in the colour of the cotton material. Concurrently, the tensile strength and elongation at break were increased, a phenomenon associated with the formation of bonds between the surface and nanoparticles. The electrical conductivity of composite textiles was found to be contingent upon the particle concentration in the modification system. It was determined that cotton modified with TiO_2_/PPy nanoparticles devoid of silicone exhibited the highest electrical conductivity. The maximum attainable electrical conductivity was observed to be 0.1 S, at a nanoparticle concentration of 10% within the system. It is important to note that the value of electrical conductivity decreases slightly after 20 washing cycles, which will allow the coating to be used for a long time [197].

Cu-based MOFs can also be used to create conductive textiles [198], MXene [199,200], as well as golden nanowires [201], graphene [202], ZnO nanoparticles [203], silver nanowires and MXene [204,205] multiple layers of silver and carbon black [206].

## 5. Stimulus-Sensitive Textile Materials

The utilisation of special dyes facilitates the creation of heat-sensitive textiles. The external factors that influence this process can result in alterations to the chemical structure of the dye, consequently modifying its characteristics. Stimuli-sensitive dyes can be activated by physical, chemical, or mechanical environmental influences or by special external signals. Modern methods allow this to be done predictably and profitably [207].

The classification of chrome phenomena resulting from the interaction of the “sensor” with external stimuli can be approached through various criteria. The stimuli encompass a range of physical phenomena, including thermochromism (the change in colour caused by temperature), photochromism (the change in colour caused by light), chemichromism (the change in colour caused by a chemical reaction), solvation chromism (the change in colour caused by moisture), ionochromism (the change in colour caused by pH), piechromism (the change in colour caused by pressure) and electrochromism (the change in colour caused by electric currents) [208].

**Figure 7:**
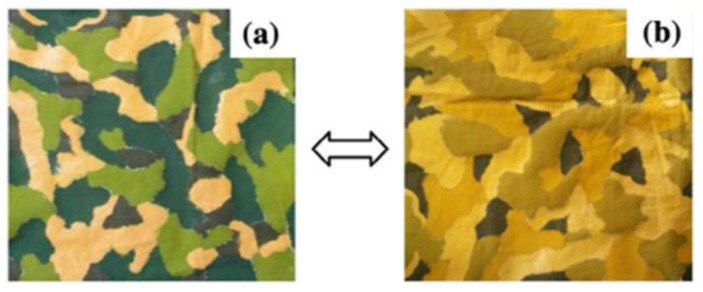
Chrome phenomena on textiles (**a**) without heating (**b**) with heating at 60 °C for 2 min [209].

As demonstrated in Figure 7, thermochromic dyes are being used with increasing frequency in the textile industry [209]. This effect can be actively exploited to create a unique design, to disguise something, or to develop sensors [210].

In the field of textile research, thermochromic dyes can be categorised into two distinct types: leuco dyes and liquid crystals. The former possesses a singular colour that undergoes a transformation during molecular rearrangement, while the latter exhibit a broader spectrum of colour variations. The primary mechanism of thermochromism in a liquid crystal system is attributable to the selective reflection of light by a liquid crystal, the molecular arrangement of which undergoes variation in accordance with temperature. In the context of chiral nematic liquid crystals, the observed colouration is contingent upon the pitch length of the spiral. The visual effect is characterised by a transition through a spectrum of colours. In the utilisation of thermochromic dyes, the employment of additional carriers is frequently observed in order to assist in the preservation of their labile structure. In order to achieve this objective, there are two possible methodologies that could be employed. Firstly, the substances could be encapsulated within microcapsules. Secondly, they could be applied to the surface of nano- and micro-particles. This renders the successful application of thermochromic dyes as an additive to traditional dyes a possibility, thus facilitating the creation of smart chromophore textile materials [211]. In certain composite systems, the color-forming agent, otherwise known as the developer, is subjected to elevated temperatures in excess of its melting point. This process ensures the interaction between the agent and the substrate, consequently inducing a color change. Subsequent to cooling and condensation, the composite reverts to its original shape and colour [212].

As a secondary principle, the molecular rearrangement of the dye groups has been identified as the second fundamental aspect of this phenomenon. This effect manifests when one of the dye components is an electron-donating compound, while the others are electron-acceptor compounds. As the temperature rises, the molecules dissociate, thereby ensuring the interaction of the two components of the dyes, which leads to a further change in the colour of the fabric. The creation of heat-sensitive textile materials can be facilitated by the utilisation of special groups of substances, such as naphthopyrans. Spirooxazines, spirolactones and other analogous compounds represent a subset of such groups [213]. Spirooxazines are defined by the presence of a spiro sp3 hybridised carbon atom, which functions to divide the molecular structure into two fragments consisting of orthogonal heterocyclic rings with non-conjugated π-systems [213].

The absorption of localized π-systems occurs in the ultraviolet range, thereby rendering the spirooxazine molecule colorless. When the oxazine (C–O) bond is broken under the influence of temperature or ultraviolet radiation, spirooxazine switches to the colour form of photomerocyanin with an open ring. The photomerocyanin molecule reverts back to the colorless molecular state of spirooxazine with a closed ring when the oxazine bridge is reformed [214]. The utilisation of inorganic materials as components of stimulus-sensitive paints is a subject that merits further consideration. An exemplar of such materials is aluminium oxide-strontium. It is important to note that due to the complexity of the methods of modification and application of inorganic agents to the surface of textiles, such agents are rarely used. Aluminium oxide-strontium [215] and doped with lanthanides (SrAl 2 O 4:Eu 2+/Dy 3+) [216] are the two forms of aluminium oxide-strontium. Aluminium oxide-strontium is an inorganic dye known as a long-lasting phosphorescent material used for colouring various materials [217].

**Figure 8:**
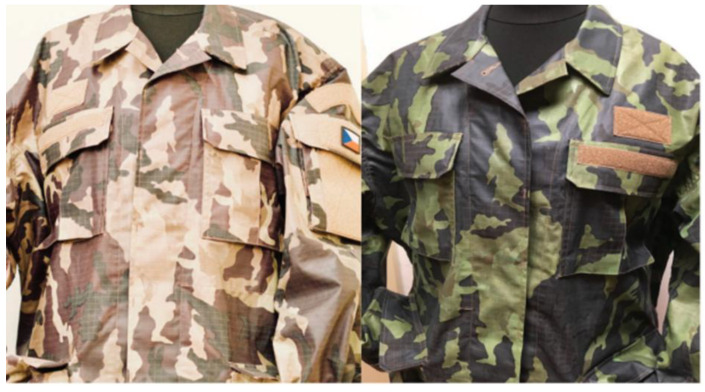
Stimulus-sensitive attire positioned on the left of the image, with the conditions to the right delineated by warmth and coldness [219].

Fluctuations in ambient temperature and body temperature can result in a wide range of colour transitions on textiles, due to the manner in which clothing is made [218]. In addition, thermal ink can be used to create camouflage clothing that is able to “adapt” to the surrounding conditions. The following paper is available at [219]. A strategy for modifying camouflage clothing using stimulus-sensitive ink is presented (Figure 8). It can be used effectively in a variety of weather conditions. Such textiles have the capacity to adapt to environmental conditions, thereby allowing them to exhibit increased levels of secrecy, depending on the prevailing temperature, for example, in sandy or wooded conditions (Table 2).

Some types of thermochromic dyes for the textile industry are shown in the table.

It has been observed that thermochromic dyes are primarily applied through direct or silkscreen printing techniques [227].

The incorporation of specialised substances that exhibit thermochromic or photochromic properties can incur a number of costs. It has been demonstrated that there is a significant decrease in breathability on the painted area in comparison with native textiles, with a reduction of up to 70% being observed. However, the light fastness of light green images remains unsatisfactory and requires further improvement. It has been demonstrated that high concentrations of thermochromic capsules can lead to a dramatic decrease in abrasion resistance. This effect is primarily attributable to the agglomeration of microcapsules of thermochromic ink that coat the surface. When employed in the context of camouflage or camouflage attire, these additives serve to augment the signal within the near-infrared spectrum. This phenomenon has the potential to adversely impact the utilisation of these substances by the end user. It is imperative to emphasise the substantial increase in cost, which will impede the large-scale production of clothing utilising stimulus-sensitive dyes.

## 6. Bioactive Textile Materials

The development of a new type of textile material that increases human safety is being driven by various global problems, including climate change, pandemics of viral diseases and pollution of the world’s oceans [62,228].

Textile materials have been demonstrated to exert a considerable influence on the propagation of diseases, with a particular emphasis on viral and bacterial pathogens [229]. The development of novel products pertaining to health and hygiene constitutes a pivotal undertaking for researchers across diverse disciplines. The accelerated mutation rate of diverse bacterial and viral strains, in conjunction with the dynamic advancements in genetics and molecular biology, has precipitated the emergence of virulent and rapidly proliferating strains [230]. At the same time, the presence of textile materials in contact with the body provides an ideal environment for the growth and reproduction of a variety of microbes, which can lead to skin and related diseases [231]. One potential method for limiting the propagation of various microbes is the creation of antibacterial materials for utilisation in clothing [232].

The preceding evidence indicates the significant importance of enhancing textile materials with the aim of curtailing the propagation of diverse diseases through the fabrication of bioactive fabrics [233,234]. The utilisation of such materials in the form of personal protective equipment within specialised institutions (e.g., masks and dressing gowns) is a potential solution during epidemics. Furthermore, these materials can be employed for the prevention of diseases.

### 6.1. Organic Bioactive Agents

Zaharia, C. et al. presented an emulsion composed of beeswax and thyme essential oils that functioned as a modifying antibacterial agent for textiles. An emulsion comprising water, glycerine, chitosan and Tween 80 as a surfactant was prepared at a temperature of 60 °C, into which molten beeswax and thyme essential oil were incorporated. The quantity of wax and essential oil in the system has been further optimised. The precursor mixture was then subjected to further processing using a high-speed homogenizer. The resulting system was an oil-in-water emulsion that was employed for the impregnation of cotton fabric. The capacity of the tissue to inhibit the growth of *E. coli* and *St. aureus* microorganisms was found to be contingent upon the presence of biologically active substances (polyphenols and flavonoids) within the emulsion. Concurrently, *St. aureus* exhibited heightened sensitivity to these effects in comparison to *E. coli*, a phenomenon that may be associated with the composition of the cell wall. When containing 3 or 4 g of beeswax and 4 mL of essential oil (per 100 mL of emulsion) in modifying formulations, cotton samples demonstrated a degree of inhibition of 59.17% against *St. aureus* and 56.11% against *E. coli* [235].

Karanikas et al. proposed the addition of polyhexanidine bisguanidine (PHMBG) to commercial pigments, specifically Disperse Blue 60:1 and CI Disperse Yellow 54:2 (Yorkshire Farben GmbH, Krefeld, Germany), for the dyeing of polyester and polyamide. It was demonstrated during the course of the work that the antimicrobial additive does not affect the colloidal state of the ink, and that it remains stable for a period of 180 days. It is noteworthy that concurrently, PHMBG increases surface tension and introduces fluctuations in the viscosity of the system, while maintaining all indicators within the acceptable limits for inkjet printing. The antibacterial additive exhibited no substantial impact on colour intensity, nor did it affect the resistance of images to washing and abrasion. An antibacterial test was used to confirm the high antibacterial activity of paints with PHMBG additives in relation to *K. peimopiae*. Concurrently, a 99.9% inhibition efficiency was attained at a concentration as low as 2%. The antimicrobial activity exhibited by the compound under investigation was found to persist even after 20 washing cycles, as evidenced by the following source: [236]. The research conducted by Karanikas, E. et al. demonstrates a method of modifying the composition of ink that does not violate the fundamental technological parameters of dye solutions. Furthermore, it is noteworthy that this modification also exhibits excellent, prolonged antibacterial activity. The simplicity of the ink modification method renders polyhexamethylene bisguanidine suitable for use in various dye formulations for inkjet printing, with the aim of creating an antibacterial coating. The primary benefit of this approach is its simplicity and the absence of complex technological processes, which will facilitate its scalability.

**Figure 9:**
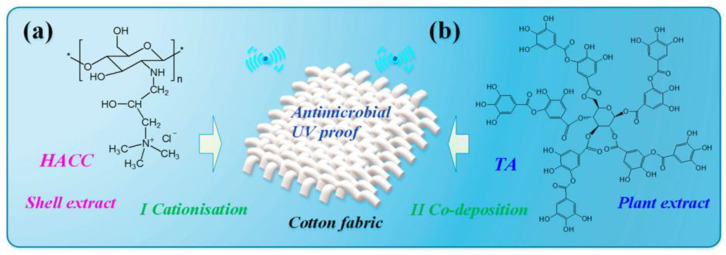
A model of chemical modification of the cotton fabric surface with (**a**) hydroxypropyltrimethyl ammonium chloride chitosan (**b**) tannin acid [237].

The surface modification of fabrics represents a successful strategy for the creation of antibacterial formulations, whilst concomitantly enhancing the intensity of staining. In a recent study, Yuyang Zhou and colleagues proposed a novel method for modifying the surface of cotton using tannin acid (TA) and hydroxypropyltrimethyl ammonium chloride chitosan (HACC) (Figure 9). The modification was achieved through a sequential adsorption process applied to the material. The utilisation of ultrasound and heating in the adsorption of HACC resulted in the surface acquiring a partial positive charge, thereby facilitating effective attachment of tannic acid. Yuyang Zhou and colleagues have conducted a study on the kinetics of substance attachment to the cotton surface, with the objective of enhancing the control of technological parameters during the production process. Utilising spectral characteristics, it was ascertained that there is no effect on the initial colour characteristics, which can have a positive effect on the aesthetic characteristics of the material. The treated tissue exhibited noteworthy antibacterial properties in relation to *E. coli*. The material demonstrated concentration-dependent toxicity, with 50% inhibition of bacteria observed at a tannic acid concentration of 2 g/L. At a concentration of 8 g/L, the surface inhibited 96% of bacteria. The potential mechanism of cytotoxic activity may be associated with the disruption of bacterial membranes, thereby limiting the diffusion of substances necessary for bacterial survival [237]. In the event of continuous surface modification, it is necessary to carry out a more detailed characterisation of the mechanical characteristics, breathability, and colorimetric characteristics of textile materials.

**Figure 10:**
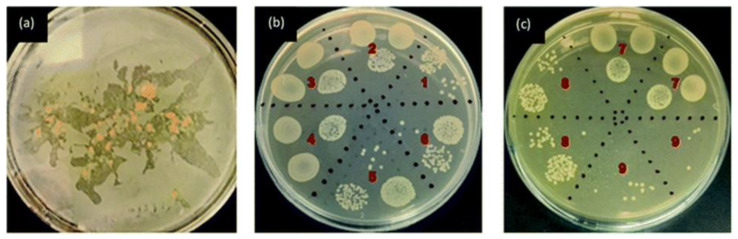
Antibacterial activity against *E. coli* (ATCC 25922) according to the ASTM E2149 Test Method for (**a**) FMN powder (**b**,**c**) chromojet printed textile [238].

Sweta Narayanan Iyer et al. posited the hypothesis that it may be possible to modify the ink composition for digital inkjet printing using flavin mononucleotide (biological flavin) (FMN). The ink was utilised for the purpose of printing on cotton fabric and polyethylene terephthalate, employing a Dimatix Sapphire QS-256/80 AAA inkjet printer (Fujifilm, Valhalla, NY, USA) with a printable drop size of 80 square metres. The addition of various concentrations of FMN had no significant effect on the rheological properties of the ink, and the necessary values for inkjet printing were retained. Untreated samples and FMN printed on PET demonstrated no bacterial activity, yet they facilitated bacterial growth (samples No. 2, 3, 4 and 7 in Figure 10). However, the application of FMN to cotton textiles (1% FMN, samples No. 5 and 6 in Figure 10) resulted in a substantial reduction in bacterial growth, indicating a promising potential for FMN as a functional additive. Furthermore, the ink proposed in the work exhibited pronounced fluorescent properties, thereby significantly expanding the material’s aesthetic potential and offering new applications for textile materials [238].

### 6.2. Creation of Bioagents Based on Inorganic Materials

It has been demonstrated that nanoparticles of various metals (metal oxides) possess pronounced antibacterial activity against various groups of microorganisms [169,239,240]. The utilisation of these properties as modifying agents in the creation of textile materials is a key consideration. The addition of nanoparticles to textiles composed of polymeric materials, either directly to the mass or as a surface-modifying coating, has been demonstrated. Inkjet printing has also been demonstrated as a method of fabricating textile materials with antibacterial properties [241]. In this instance, antibacterial components can be incorporated into the ink composition or included in the pre-coating for the material that is utilised as a substrate for inkjet printing [242]. The high antibacterial properties obtained by inkjet printing using silver nanoparticles were considered in the work [243].

In the presentation, Farheen Afzal put forward a strategy for modifying cotton textile materials using zinc oxide nanopowder (ZnONPs). In addition, ZnONPs were modified with 3-glycidoxypropyltrimethoxysilane, which facilitated interaction with cellulose under alkaline conditions. In this regard, cotton was immersed in a 1% sodium hydroxide solution, and then the fabric was immersed in an aqueous dispersion of ZnONPs, pressed and dried at elevated temperature. The formation of a covalent bond between zinc nanoparticles has been demonstrated to ensure a high degree of resistance to external influences, such as washing. This assertion is supported by electron microscopy data. The tissues exhibited significant antibacterial activity against *S. aureus* and *E. coli*, with the active forms of oxygen present in the tissues modified with zinc oxide nanoparticles proving effective in inhibiting up to 99% of *S. aureus*. The observed discrepancy in biological activity may be attributable to the divergent chemical composition exhibited by Gram-positive and Gram-negative bacteria. The tissue was also found to be effective against the dengue virus and the hepatitis C virus (HCV). It is notable that the material retained its effectiveness even after 30 washings, a property that can be caused by the occurrence of covalent interactions between the cellulose present in cotton and the epoxy group sewn to zinc oxide nanoparticles [244].

**Figure 11:**
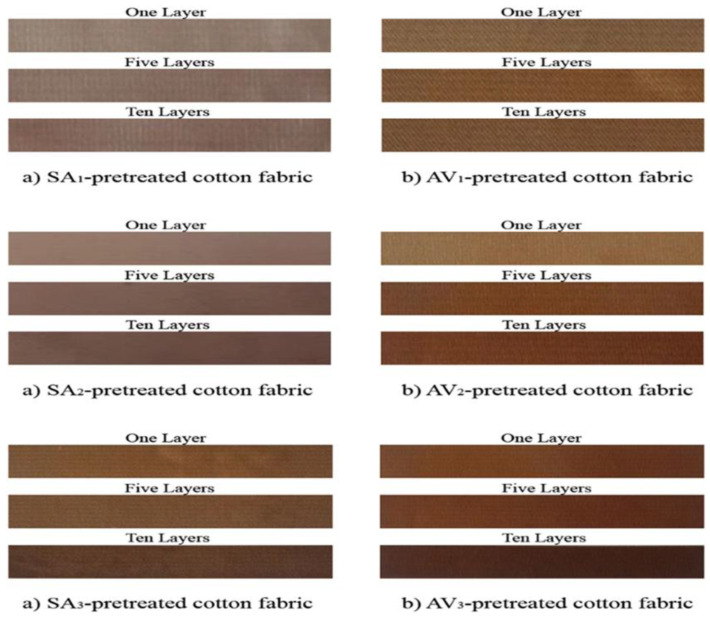
Illustrations of lines printed with water-based ink in one pass (one layer), five passes (five layers) and ten passes (ten layers) on cotton fabric, pre-treated with (**a**) sodium alginate and (**b**) aloe vera extract [245].

An unconventional technique for fabricating an antibacterial coating on cotton was employed by Atasheh Soleimani-Gorgani et al [245]. In their study, they proposed a novel in situ method for synthesising nanoparticles using aloe vera extract (AV) and sodium alginate (SA). To this end, cotton samples were initially modified with modifying compounds, followed by the application of a silver nitrate solution. The study demonstrated that sodium alginate exhibited no impact on the colouration of cotton. Conversely, aloe vera extract was observed to enhance the yellow hue of the surface, a phenomenon that has the potential to adversely impact the product’s aesthetic appeal. Electron microscopy data has demonstrated that polymers facilitate the smoothing of the native cotton surface; however, concurrently, an augmentation in the concentration of aloe vera has been observed to result in the formation of bumps and an increase in surface relief. The application of spectrophotometric image evaluation revealed that any preliminary modification significantly enhances ink adhesion. Furthermore, an increase in the number of layers results in a more even deposition of nanoparticles on the surface (Figure 11). This method produces silver nanoparticles with a size range of 30 to 60 nm. The size of the coating was found to be contingent upon the concentration of the pre-coating and the number of layers applied to the surface of the material. Cotton with printed images using silver nanoparticles has demonstrated antimicrobial activity against *S. aureus*, *E. coli* and *C. albicans*, which depended on the number of layers (concentrations of particles on the surface). Concurrently, the antibacterial activity exhibited by the pre-coated SA material was found to exceed that of the AV material, despite the latter comprising an equivalent number of layers. In addition to samples with printed silver nanoparticles and pre-coated with SA, it was demonstrated that antibacterial activity could be sustained even after five washing cycles had been completed. Furthermore, the AV inhibition zones exhibited a decrease in the samples. In the course of the research, a unique methodology was proposed by the authors, which sought to facilitate the interaction of silver nitrate and reducing agents on the surface of tissue in order to produce antibacterial AgNP. The interaction of ink and reducing agents in the pre-coating process has resulted in the creation of a unique material, which exhibits high antibacterial properties against various groups of microorganisms.

In the research conducted by Xuexin Wang and colleagues, silicon dioxide nanoparticles were modified with CTAB and glycidoxypropyltrimethoxysilane. The findings of this study demonstrated that the cotton samples modified with nanoparticles exhibited enhanced image quality, attributable to the formation of ionic bonds between the modified SiO_2_ particles and the anionic pigment inks. This research is further supported by the following publication: [246]. The resulting material exhibited antibacterial activity against *S. aureus* and *E. coli*, which is attributable to the interaction of CTAB and bacterial membranes. The antibacterial activity of the material was found to be contingent upon the concentrations of surfactant within the system.

This section demonstrates the extensive potential for the development of novel materials that exhibit a specific biological activity. The advent of sophisticated technologies has rendered feasible the fabrication of materials that exert a general toxic effect on all bacteria, as well as on specific bacterial groups (e.g., Gram-positive bacteria). The employment of nanoparticles that demonstrate toxicity at comparatively lower concentrations, whilst retaining the mechanical properties and breathability of the original material, is of particular relevance in these methods. It is important to acknowledge the restricted application of nanotechnology in industrial textile technologies, despite the simplicity of creating and modifying substances and the high efficiency that can be achieved. This phenomenon may be associated with concerns regarding the stability and long-term effectiveness of nanotechnology agents, as well as research demonstrating the potential for successful scaling up to large volumes. It is also important to note that nanoparticles in high concentrations can have a toxic effect on humans, which can reduce the biosafety of textile industries.

## 7. Quality Control of Textile Materials

A plethora of standards are currently in existence that can be utilised for the purpose of regulating specific characteristics of textiles [247]. These standards encompass a wide range of parameters, including colour characteristics, mechanical strength, steam and water permeability, resistance to abrasion and other mechanical influences. It is important to note that the majority of the methods are specific to a particular type of fabric and are not universally applicable. This limitation precludes the possibility of a direct comparison of the characteristics of different materials [248,249,250,251].

A classification system can be established that divides all characterisation methods into several groups according to the type of parameter being determined.

1. Aesthetic;
  - colorimetric characteristics;
  - gloss (if present);
  - uniformity of color/morphology of the surface.
2. Comfort;
  - breathability;
  - softness;
  - moisture/vapor permeability;
  - heat preservation.
3. Durability (resistance);
  - resistance to washing;
  - resistance to dry/wet friction;
  - resistance to the effects of physiological fluids;
  - mechanical strength (tensile, tensile, bending, etc.);
  - resistance to light.
4. Functionality
  - biological activity;
  - resistance to water (rain, liquid column);
  - interaction with electric current;
  - resistance to extreme temperatures.
5. Defective (presence of damage)

The amalgamation of these characteristics enables the comprehensive determination of the properties of a given material. A comprehensive understanding of the potential applications of specific textile materials or products can be formed by analysing all the key parameters. In order to study the proposed indicators, both standard methods and alternative modern methods can be used. The standard methods are prescribed and registered in official documents, while the alternative modern methods have been developed by researchers. The methods employed are intended to ensure the veracity and replicability of the data [252]. The method’s pertinence is contingent upon the feasibility of analysing textiles of diverse provenance, a condition that facilitates a comparative examination of fabrics [253].

**Figure 12:**
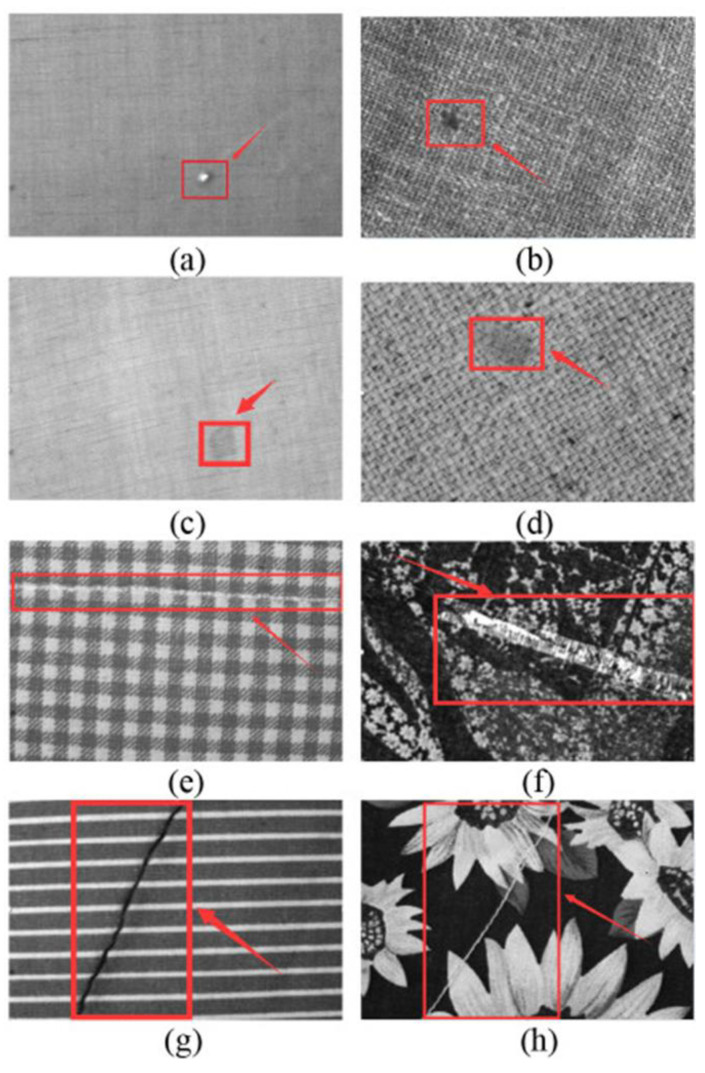
Images of model fabrics with defects. Dataset contains four defects, (**a**,**b)** hole, (**c**,**d**) spot, (**e**,**f**) wire, (**g**,**h**) dark thread [254].

An exemplar of such methodologies is provided by the research conducted by Huosheng Xie, which employs artificial neural networks to detect tissue defects (Figure 12) [254]. The standard Single Shot MultiBox Detector (SSD) model has been enhanced by the researchers, a development that has resulted in a significant enhancement in the speed of detection. The Fully Convolutional Squeeze-and-Excitation (FCSE) model has been integrated into the system, with the objective of enhancing the accuracy of the analysis and reducing the number of errors. It is evident that the optimisation of the number of cells has a substantial impact on the model’s capacity to accurately detect defects in the form of long stripes. The analysis is facilitated by the utilisation of images of fabrics, which can be produced in real time to ascertain defects in production and to monitor the quality of finished products. The experimental data obtained has confirmed the high speed and accuracy of defect detection on fabrics with different textures. The proposed approaches have the potential to be integrated into technological schemes and utilised for the identification of defects and damage on textiles. The utilisation of machine learning and neural networks is the subject of a comprehensive study in the Peiyao Guo review [255].

Another illustration of a universal methodology is the principle of porosity determination, as proposed by Mateusz Kowalski et al. [256]. This methodology is designed for application in the analysis of digital images with varying magnifications, wherein individual pores are evaluated in two perpendicular directions. The lumens (pore parameters) were determined using digital image analysis. The average pore size was estimated using the Motic Images Plus 2.0 ML program. Porosity was defined as the ratio of the through-hole area to the total area analysed.

**Figure 13:**
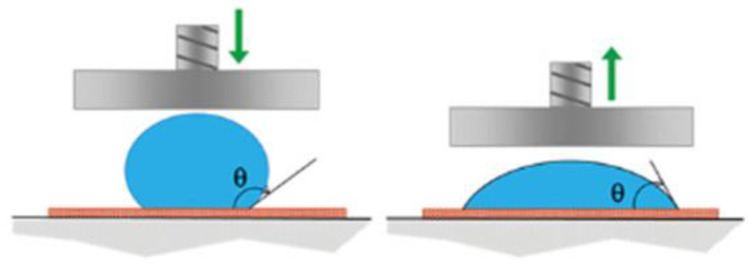
Diagram of the pressure wetting edge angle analysis test [257].

As demonstrated in the work of Safi Melki et al. [257], modified approaches for the analysis of hydrophobic properties and the edge angle of wetting on textile materials have been demonstrated (Figure 13). The “forced wetting” method was founded on the Cassie-Baxter/Wenzel transition [258] and had previously been utilised as an alternative for the edge angle of wetting on a rough surface. The proposed method entailed the application of a drop to a hydrophobic surface and the subsequent compression of this drop at a certain pressure (1 or 10 kPa). This was followed by the release of pressure and the measurement of the wetting edge angle. The study demonstrates that the samples possess approximately the same wetting edge angle; however, the dynamic parameters of the rolling angle and the “forced wetting” tests demonstrate a significant difference between pile fabrics and cotton textiles, which could not be controlled using traditional wetting tests. The proposed method of “forced wetting” facilitates a comprehensive evaluation and comparison of the hydrophobic properties of textile materials.

In the present study, a novel methodology was proposed for the purpose of determining the colour characteristics of cotton and fabrics [259]. The fundamental principle of the method is an independent examination of the colour characteristics by twelve judges. Each judge performed a colour vision diagnosis using a simple test (Ishihara), and all exhibited normal or adjusted to normal vision (Table 3). The judges were instructed to group the samples according to the similarity of image quality, so that each group had some common features that set them apart from other groups. The judges were permitted to form as many groups as they deemed appropriate. Following the completion of the grouping process, a rating was assigned to each group, reflecting the assessors’ perception of the quality of the samples in each group. Finally, the observers were invited to provide a rating number at or above which they would consider the samples acceptable for purchase or use. Each judge operated within two distinct sessions, utilising disparate images. Illustrative examples of this approach included the presentation of paintings depicting silverware and a portrait of a girl. The present paper considers this method for assessing the quality of ink for inkjet printing, while the approach proposed by the authors can be expanded to evaluate the colour characteristics of the fabric, images applied to it, or to assess the effect of modification conditions on the colorimetric characteristics of the product.

The following table presents a range of universal methodologies that can be employed for the comparative analysis of indicators from diverse tissue samples.

## 8. Prospects and Challenges

### 8.1. Economic Considerations

Given the high-volume nature of textile production, economic viability is paramount. Functionalization inherently increases costs through process adaptation, precursor procurement, and additional quality control. For industrial implementation, strategies must be evaluated through comprehensive techno-economic and scalability analyses. Achieving multifunctionality without compromising cost-efficiency would be decisive for technology transfer and large-scale adoption.

### 8.2. Mechanical Robustness

Physical and chemical treatments required for imparting functionality frequently compromise tensile strength and wear resistance, limiting service life. Establishing quantitative relationships between processing parameters and mechanical properties at the design stage will be essential to reconcile performance with durability, thereby accelerating industrial translation.

### 8.3. Air Permeability

Deposition of functional coatings or incorporation of modifiers may result in fiber thickening and surface coverage, thereby reducing air permeability. Such modifications compromise thermal regulation and diminish wearer comfort, which are crucial attributes for everyday textiles. Establishing a quantitative correlation between processing conditions and permeability would provide a methodological basis for engineering industrial technologies that retain comfort while enhancing fabric functionality.

### 8.4. Wash Fastness

Since daily use implies repeated laundering, the preservation of functionality under washing conditions is of paramount importance. Standardized testing should demonstrate retention of functional properties for at least 50 wash cycles to ensure practical applicability. High wash fastness would expand the prospects for employing functionalized textiles not only in specialized protective clothing but also in mass-market apparel.

### 8.5. Environmental and Toxicological Considerations

The ecological footprint and toxicological safety of modifying agents remain underexplored. While a substantial portion of conventional modifiers are well-characterized in terms of biocompatibility and environmental impact, the increasing utilization of nanomaterials as functionalizing agents introduces new uncertainties, as their long-term safety profiles are insufficiently investigated. Given that industrial production targets consumer goods with daily contact, precursors must be strictly non-toxic and safe for both manufacturers and end-users. In parallel, comprehensive studies on degradation pathways, recycling routes, and disposal strategies should be undertaken, enabling integration of functionalization processes into sustainable industrial frameworks.

### 8.6. Aesthetic and Colorimetric Attributes

Although multiple functionalization techniques, some of which provide multifunctionality, have been proposed, their impact on visual and aesthetic parameters remains largely overlooked. Surface modifications and post-treatments may substantially alter color, gloss, and other visual properties. Considering the growing significance of aesthetics in consumer choice, systematic evaluation of colorimetric and appearance-related parameters is essential for bridging technological innovation with market acceptance.

## 9. Conclusions

The advancement of functional textiles offers tangible potential for improving quality of life. The purpose of this article was to demonstrate promising trends in the creation of functional textiles. Approaches for producing conductive textile patterns on fabrics of various origins are demonstrated. The paper presents simple methods for using inkjet printing on various textile materials. The possibilities of creating bioactive textiles are explored. Furthermore, the article discusses modern techniques that can significantly improve the properties of textile materials, thereby expanding their functionality. These approaches may serve as a foundation for the development of specialized garments and personal protective equipment.

Equally important is that we addressed current methodologies for assessing textile quality, including those applicable to functionalized fabrics. Whereas most conventional techniques remain material-specific, our review emphasizes both established and emerging approaches that are adaptable to a broader spectrum of textile systems.

At present, large-scale industrial integration of functionalization remains limited, restraining the growth of the sector. Nevertheless, the surveyed strategies underscore significant opportunities for the creation of multifunctional materials. With this article, we would like to highlight the importance of integrating new innovative methods into textile materials.

## Figures and Tables

**Table 1:** Main modifying agents used for natural fibres.

№	Modifying Agent	Standard Conditions	Basic Purpose	References
1	Hydrogen peroxide	The subject should be immersed in the solution for a period of time ranging from several minutes to several hours. In certain instances, the application of heat may be employed.	Adjusting morphology and increasing fibre roughness and whiteness.	[96,97]
2	Polylactic acid	Acid melt impregnation of fibres.	Increased strength, improved hydrophilicity.	[98,99]
3	Organosilanes	Soaking in a silane solution, or applying directly to the fibres without the use of solvents. In addition, heating can be used	Thermal resistance, hydrophobicity	[100,101]
4	Alkali	Treatment of fibres at room or increased temperature for several hours	Surface hydrophilisation, increased roughness.	[102,103]
5	Benzene, benzoyl chloride and their derivatives	The treatment is carried out for a few minutes under room conditions or under heat. Could be pre-treated with alkalis	Improved mechanical, thermal and ductility properties	[103,104]
6	Methacrylate and its derivatives	Maintain for several hours at elevated temperature. For chemical grafting a process initiator can be additionally used	Customisation of surface properties and mechanical properties	[105,106]
7	Potassium permanganate/ potassium dichromate	Treatment for 1–15 min under indoor conditions	The increased wettability and hydrophilicity of the surface have been demonstrated, as well as its use in adjusting surface roughness. This may result in a substantial decrease in strength characteristics	[107,108]
8	Acetic anhydride	Processing is carried out at elevated temperature in an acidic environment	Strength, mechanical, thermal properties. Hydrophobic properties can be achieved for some fibres.	[109,110]
9	Sodium chlorite	Treatment for several hours at an increased temperature	Improvement of mechanical, strength and thermal properties. Reduction in cellulose crystallinity (if cellulose fractions are present). Can be used as a whitening component.	[110,111]

**Table 2:** Of stimulus-sensitive agents for textile materials.

№	Dye/Pigment	Material	Temperature Transition	Colour Transition	References
1	Particle’s core/shell 0.31 g crystal violet lactone, 0.94 g bisphenol A and 18.75 g of 1-tetradecanol were dissolved at 200 °C in thermostatic oil. The bath was soaked for 1–2 min, after which a clear and homogeneous oil was obtained.	Fabric	25–45 °C	blue/gray—reversible	[220]
2	thermochromic ink produced by Matsui Shikiso Chemical Co., Ltd. and L J Specialties Ltd. containing water, glycerin, Akramin B.A. (binder, Bayer, Germany), ammonia	The textile fabric contained a mixture of polyester and cotton. The fiber composition is 65%/35% and its thickness is 0.34 0.3 mm.	30–39 °C	6 colours	[219]
3	Bromocresol violet, boric acid and tetradecyl alcohol. It was stirred at a temperature of 60 ° C for 10 min, and then placed in a silicon sol for 60 min.	100% cotton	between 25 and 50 °C for 1 min	reversible yellow and pink transition, which was determined by impurities in the system	[221]
4	taking 4 g of carbamide, 20 g of sodium alginate and 12 g of sodium chloride and added to a glass with a capacity of 100 mL of distilled water with constant stirring. Thermochromic dyes (blue and orange) were supplied by the company Americos Industries Inc.	100% cotton	up to 50 °C	different colors, yellow, green, sandy	[209]
5	Licrotherm TCP 1001, TCP 1004, TCP 1005 and TCP 1006 microcapsulated liquid crystals dispersed in an aqueous acrylic emulsion	on black nylon/lycra fabric (Guildford Europe) on a flat sole	27–32 °C	Different colours	[222]
6	Special FX Creative (Newhaven, UK) water-based TC dispersion yellow (TCy), Magenta (TCm), Blue (TCb) and Black (TCk)	with 74% polyamide and 26% elastane rubber bands with a width of 20 mm, supplied by the company Nastrotex—Indústria de Passamanarias Lda	31 °C	Different colours	[223]
7	Linseed oil Alkyd resin Thermochromic microcapsules Auxiliary substances	Fabric, paper	40 °C	blue—grey	[224]
8	microcapsules in PVA film	polyvinyl alcohol films	20–48 °C	yellow to pink	[225]
9	synthesized particles with zirconium dioxide	cotton fabric	Beginning with 25 °C	yellow to grey	[226]

**Table 3:** Methods of characterization of textile materials.

№	Control Method	Description	Material	References
1	Colour rendering/Colour intensity	The determination of this parameter is possible in the case of printed and dyed fabrics. The Kubelka–Munk equation (K/S) is utilised, with the R-reflection coefficient being the variable of calculation.	Fabric/any textile	[260]
2	Colour rendering	The K/S value was determined by utilising a Datacolor 650 colorimeter and a D65 light source with a 10° viewing angle. The printed sample was subjected to meticulous measurement. The colour rendering of the sample was measured in order to ascertain its chromatic properties. The visible spectrum, which is defined as the range of electromagnetic radiation with a wavelength between 400 nm and 700 nm, is analysed using the Kubelka–Munk theory.	Hydroentangling nonwoven material 89 g/m2. polyethylene terephthalate (PET)	[261]
3	Line width, blurriness and rough edges, raggedness, sharpness, modulation and ink spreading	It is monitored visually or using a ruler, micrometer, microscope or other measuring device. For this purpose, a special target can be printed with a thickness (1/8, 1/4, 1/2 and 1 pt) both horizontally and vertically. Vertical orientation is used to determine the minimum achievable thickness of image details.	Polyester	[262]
4	Marginal wetting angle	The wetting edge angle can be measured using specialised equipment (e.g., KRUSS), a microscope or a high-speed camera. This can be done with both hydrophilic (e.g., water) and hydrophobic liquids (e.g., diiodomethane, 2-octanol).	Hydroentangling nonwoven material 89 g/m2. polyethylene terephthalate (PET).	[261]
5	The minimum angle required for a drop of liquid to roll off a surface.	This can be carried out using either hydrophilic liquids, such as water, or hydrophobic liquids, such as diiodomethane or octanol.	Polypropylene	[263]
6	Absorption rate	The absorption rate was measured by the height, in millimetres, that liquids rose to after 5 min. Tests were carried out using distilled water (hydrophilic) and 2-octanol (hydrophobic). Additional photos can be taken to control the speed of propagation. The ratio of these two measurements can also be analysed, with (W/O) being used as an indicator of hydrophilicity compared to the hydrophobic (oleophilic) nature of fabrics.	Polyester fabrics	[264]
7	Fabric absorbency	The objective of the experiment is to ascertain the time it takes for a tissue to absorb a fixed amount of fluid. It has been demonstrated that an increase in the duration of the absorption process results in a reduction in liquid-substrate affinity.	Cotton fabrics	[265]
8	Pressure testing of the sample in contact with PVC	The sample is to be placed on a polyvinyl chloride (PVC) sheet, which is then to be covered with a sheet of dry white filter paper. The sheet is then to be placed between two glass plates. The utilisation of glass plates is imperative in this context. The pressure is generated by the weight of the upper glass plate. The placement of multiple samples is prohibited. The resulting sample is then placed in a heating cabinet. Following a period of 72 h, the sample is retrieved from the heating cabinet. A square PVC sheet and filter paper are then separated and examined for any stains or traces left by the fabric. The PVC sheet is first examined on a white background and then on a black one.	Fabric.	[266]
9	Exposure of the sample to aggressive media alkaline/acid sweat	The samples should be excised (utilising the D2 (washing) exemplar) (see Table 1). Thereafter, they should be weighed and placed in a Petri dish. Subsequently, the freshly prepared sweat solution should be added and the mixture left to soak for 30 min at room temperature. The next step is to remove the sample, and then to scrape off the excess sweat using two glass sticks. The sample should then be weighed again. The component should be positioned between the sweat stand and the pressure plate. Further examples are provided below: The object should be positioned on the uppermost surface. The initial step in the process is to arrange the components in a superimposed configuration. Subsequently, the weight is to be positioned on the spring pressure plate, which is then to be secured in place. The weight is then to be removed, and the excess sweat is to be poured out, thus forming the desired structure. The combined test body should then be placed in a drying cabinet at a temperature of T (37 ± 2) °C for a period of 4 h.	Fabric	[266]
10	Wet and dry friction testing of fabric by grinding head	Dry: Put a piece of cleaning cloth (50 × 50) mm on the cleaning head (T = (20 ± 2) °C, humidity 65% ± 4%), humidity control for >4 h. The direction of the fabric should match the direction of movement of the friction head. Adjust the speed of the grinding head by one cycle of reciprocating friction per second (10 cycles of friction). The friction stroke on the sample = (104 ± 3) mm. The direction of the applied force is vertically downward. Force = (9 ± 0.2) N. After completing 10 cycles, remove the fabric, adjust the humidity (more than 4 h) and remove any excess fibers on the fabric that may affect performance.	Fabric	[266]
11	Fabric defect	The utilisation of a thermal imaging camera facilitates the capture of digital images of the fabric. These images are then subjected to analysis using a bespoke software program, which employs a specialised algorithm to evaluate the contrast present within the image. This process enables the identification and measurement of any defects present within the fabric	Fabric	[267]
12	Antibacterial activity	The estimation of the number of surviving bacterial cells cultured together with the textile material is a key component of the process. The analysis of cell viability can be facilitated by the utilisation of optical or luminescent spectroscopy.	Cotton	[268]
13	ζ-(Zeta) potential	The zeta potential of the surface of any textile material is evaluated. This methodology can be employed to evaluate a range of properties, including hydrophobicity and dirt resistance.	Fabric	[269]

## Data Availability

The data that support the findings of this study are available from the corresponding author upon reasonable request.
